# HealtheDataLab – a cloud computing solution for data science and advanced analytics in healthcare with application to predicting multi-center pediatric readmissions

**DOI:** 10.1186/s12911-020-01153-7

**Published:** 2020-06-19

**Authors:** Louis Ehwerhemuepha, Gary Gasperino, Nathaniel Bischoff, Sharief Taraman, Anthony Chang, William Feaster

**Affiliations:** 1 CHOC Children’s Hospital, Orange, CA 92868 USA; 2 The Sharon Disney Lund Medical Intelligence and Innovation Institute (MI3), Orange, USA; 3 Chapman University School of Computational and Data Science, Orange, California USA; 4 Cerner Corporation, Kansas, MO USA; 5 Department of Pediatrics, University of California-Irvine, School of Medicine, Irvine, USA

**Keywords:** Pediatric hospital readmissions, Cloud computing, Amazon web Services

## Abstract

**Background:**

There is a shortage of medical informatics and data science platforms using cloud computing on electronic medical record (EMR) data, and with computing capacity for analyzing big data. We implemented, described, and applied a cloud computing solution utilizing the fast health interoperability resources (FHIR) standardization and state-of-the-art parallel distributed computing platform for advanced analytics.

**Methods:**

We utilized the architecture of the modern predictive analytics platform called Cerner® HealtheDataLab and described the suite of cloud computing services and Apache Projects that it relies on. We validated the platform by replicating and improving on a previous single pediatric institution study/model on readmission and developing a multi-center model of all-cause readmission for pediatric-age patients using the Cerner® Health Facts Deidentified Database (now updated and referred to as the Cerner Real World Data). We retrieved a subset of 1.4 million pediatric encounters consisting of 48 hospitals’ data on pediatric encounters in the database based on a priori inclusion criteria. We built and analyzed corresponding random forest and multilayer perceptron (MLP) neural network models using HealtheDataLab.

**Results:**

Using the HealtheDataLab platform, we developed a random forest model and multi-layer perceptron model with AUC of 0.8446 (0.8444, 0.8447) and 0.8451 (0.8449, 0.8453) respectively. We showed the distribution in model performance across hospitals and identified a set of novel variables under previous resource utilization and generic medications that may be used to improve existing readmission models.

**Conclusion:**

Our results suggest that high performance, elastic cloud computing infrastructures such as the platform presented here can be used for the development of highly predictive models on EMR data in a secure and robust environment. This in turn can lead to new clinical insights/discoveries.

## Background

The application of predictive analytics and artificial intelligence (AI) in healthcare is met with several challenges including data access, standardization, collaboration, computing resource needs, and deployment of predictive models [1, 2]. In addition, challenges peculiar to analyses of big data must be addressed. On the one hand, the recent proliferation of big data in medicine [3] and improvements in high performance cloud computing [4] have necessitated the consideration of cloud computing for both storage and maintenance of data in medical/life science research as well as for business intelligence in general [5, 6]. The move from non-cloud to cloud technologies is further strengthened by reduction in overall cost and maintenance as well as the elasticity that cloud architectures provide. But comparisons between cloud and non-cloud storage and maintenance of big data is nuanced [7]. On the other hand, big data analyses require algorithms adapted to high performance parallel distributed computing. This has been addressed in the development and application of new methods in bioinformatics, statistical genetics, and in data science. These applications include analysis of structured data as well as medical images and genomic data [6, 8–10].

In healthcare, research and business intelligence teams can directly leverage the tools provided by cloud computing using Amazon Web Services, Google Cloud Platform, and Microsoft Azure among others. However, expertise in high performance parallel distributed computing is required to properly manage and leverage cloud computing resources on specific projects and, more so, at scale. In this study, we seek to address 3 questions as follows: First, can a high-performance distributed cloud computing tool for storage and analyses of electronic medical record (EMR) data be developed? Second, can the tool be integrated directly with the EMR and receive automated updates from the EMR? And third, can such system help in the improvement of existing machine and artificial intelligence learning models that are deployable as decision support tools? This work seeks to address these questions with an application to predicting hospital readmissions using a large multicenter data. The answer to the first question is clearly “yes” as previous studies have leveraged high-performance distributed cloud computing tools for analyses of big data in biomedical informatics and medicine [6, 11–15]. HealtheDataLab was developed by Cerner® Corporation, partially in response to the second question. It is a cloud-based high-performance parallel distributed (and elastic) tool for automated retrieval of data from the EMR, storage and management of the data, and application of machine learning and artificial intelligence tools.

We addressed the challenges of improving predictive analytics and AI in healthcare using the Cerner® HealtheDataLab platform/tool. The platform solves most of the challenges of data science in healthcare while simplifying development of large-scale predictive analytics solutions. It uses the Amazon Web Services (AWS) Simple Storage Solution (S3) bucket [11] as an encrypted data lake for storage of structured and unstructured data, and Amazon Web Services’ Elastic MapReduce for high performance distributed computing. It integrates several Apache Project Solutions, such as Hive [16] and Spark [17, 18], and uses Jupyter [19] as the integrated development environment. The platform is compliant with Health Insurance Portability and Accountability Act of 1996 (HIPAA) [20] with end-to-end encryption. Herein, the platform is utilized to provide methodological improvement to a previous study on unplanned readmissions at a single pediatric institution [21], and for developing a multi-center model for predicting general all-cause 30-day readmissions [21–25] among pediatric-age patients (patients less than 18 years) using the Cerner(R) Health Facts Deidentified Database which has recently been updated and renamed as the Cerner Real World Data. Previous research in the application of machine learning in medicine has predominantly been in adult medicine. As a result, there is need for research in the application of machine learning algorithms in pediatrics. We describe the HealtheDataLab platform and AWS tools it depends on and application to development of multi-center pediatric models for readmission using the large protected deidentified database called the Health Facts Database.

## Methods

### The HealtheDataLab platform

**Fig. 1:**
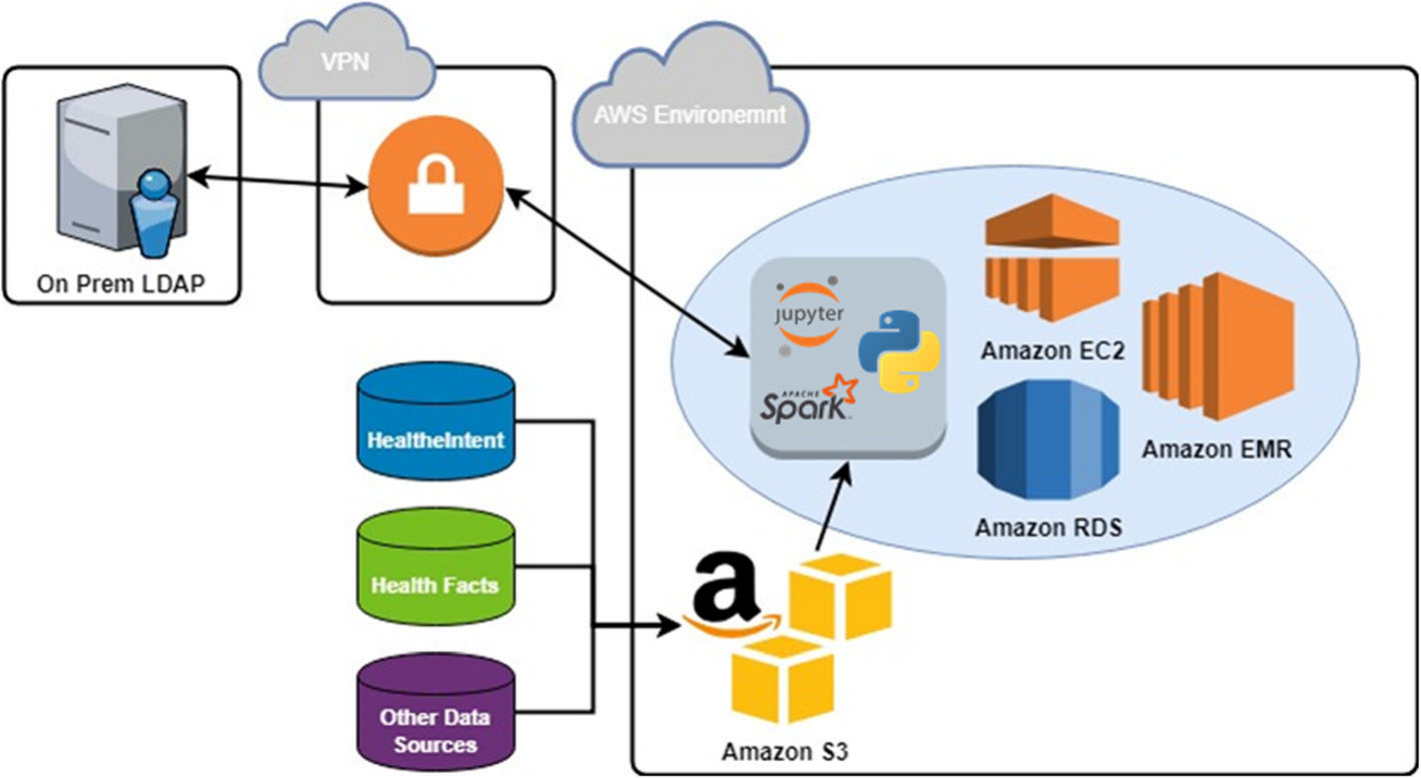
The HealtheDataLab architecture

HealtheDataLab is a data science environment designed to assist researchers and data scientists build statistical and machine learning models in an elastic, cloud-based, high-performance computing system that is HIPAA compliant. It provides users access to a wide array of data science-oriented tools for extraction, transformation, and loading of data as well as development of complex prediction models. Jupyter™ notebooks [19, 26], the Python programming language [27], and Apache Spark [17, 18] are the default programming tools accessible within HealtheDataLab, with support for the R Statistical Computing Language just added. The architecture of the platform is shown in Fig. 1.

The Jupyter notebook provides a “web-based application suitable for capturing the whole computation process: developing, documenting, and executing code, as well as communicating the results” [26] and can be shared among multiple collaborating users. The backend computing engine is built on Apache Spark, a unified analytics engine for big data analysis and machine learning [17] with a default integration with Python 3, an object-oriented programming language. Together, the Spark and Python integration is called PySpark [28].

Utilization of Amazon Web Services (AWS) [11] allows for scalability and elasticity. At the time of writing, there are 10 different HIPAA compliant services within AWS [29] that are leveraged in the deployment of a HealtheDataLab environment. These services include Amazon Elastic Compute Cloud (EC2), Simple Storage Solution (S3), Relational Database Service (RDS), Cloud Formation, Directory Service, Virtual Private Cloud (VPC), Route 53, Simple Notification Service, and Data Pipeline. Refer to Additional file 1 for details on these services. HealtheDataLab uses Cerner’s HealtheIntent platform as its primary data source and as such can receive automated data updates.

### The HealtheIntent platform

HealtheIntent is a cloud-based population health platform designed to collect data from multiple sources including EMR and used to stratify subpopulations that require targeted care. This enables healthcare systems to aggregate, transform, and reconcile longitudinal data of their members and understanding gaps in their care [30, 31]. The HealtheIntent data provided can be de-identified or identified, depending on IRB approvals for HealtheDataLab users. HealtheIntent de-identification method follows HIPAA compliant standards (Safe-Harbor Method [32]), which includes removing fields such as patient name, telephone number, e-mail address, and social security number. Date-shifting within HealtheDataLab follows Cerner’s i2b2 date shifting guide-lines by shifting dates consistently across patient records. This preserves the day of the week that the observation occurred on as well as its seasonality.

EMR data is made available in HealtheDataLab using HealtheIntent in one of two formats: the HealtheIntent Core Information Model or HL7’s FHIR® data model [33]. The HealtheIntent Core Information Model is a representation of how the data is ingested from the EHR into HealtheIntent and its implementation may vary by data source [30]. HL7’s FHIR specifications provide a “standard for exchanging healthcare information electronically,” which has been widely adopted throughout the industry [33, 34]. Both standards allow for the normalization of the data within HealtheDataLab, allowing researchers to refer to a data model and its specifications in their research. In addition to HealtheIntent data, it is also possible to upload external sources of data to incorporate into analysis, although these data sources may not be normalized to the HealtheIntent Core Information Model or FHIR data model standards. The single center models described in this study were carried out using the default EMR data in HealtheDataLab of the corresponding author’s institution.

### Cerner health facts database (Cerner real world data)

The Cerner Health Facts Database is designed to provide access to a large volume of multi-center, de-identified, clinical data. The database captures and stores information generated by Cerner Corporation which is aggregated and organized into consumable datasets to facilitate research and reporting. The data is encrypted and secured to maintain patient confidentiality and ensure compliance with HIPAA privacy regulations. It consists of clinical database tables with data on patient demographics, encounters, medications, laboratory tests, clinical events, and diagnoses among others. In 2018, it consisted of greater than 95 healthcare systems comprised of greater than 650 individual facilities within the United States that are clients of Cerner Corporation who contribute de-identified copies of EMR data. At the time of this study, the Health Facts Database consisted of 6.9 million patients and 503.8 million encounters across all care settings. The multi-center models developed in this study were built using a subset of data from the database based on a priori inclusion criteria. As at the first quarter of 2020, the format of the database was been changed to include the HealtheIntent Core Information Model with additional updates and now referred to as the Cerner Real World Data. This update was implemented after model development for this study. As a result, this study used the last iteration of the Health Facts DB.

### Data

Replication of a single-center model on 30-day unplanned readmission [21], herein referred to as M0, was performed to validate the HealtheDataLab platform by replicating and comparing to previously performed work within our institution. Improvements to M0 were obtained by addressing a methodological limitation of model M0 (the exclusion of encounters for chemotherapy); increasing the sample size by considering a longer period of admission; and exploring additional EMR data such as medications made more readily accessible in HealtheDataLab. This led to the development of an updated single-center model for unplanned readmission, referred to as M1 from here on. Furthermore, we explored the generalizability of the methodological approach and results we obtained on the single institution models to the multi-center database. We approached this by building a model, M2, considering all readmissions (planned and unplanned) on the single center data to allow for a more precise comparison with models of all readmissions on the multi-center database, M3. Note that models M1 and M2 were built using copies of our HealtheIntent EMR data made available in HealtheDataLab. Model M3 was built using the deidentified multi-center Health Facts database which does not include information required to separate planned from unplanned readmissions. We used the same statistical and machine learning methodology for development of readmission models M1, M2, and M3. Models M1 and M2 were random forest models; however, for model M3, we present both a random forest and multi-layer perceptron (MLP) version. The data used to develop the single institution models were not included in the data of multi-center models. Details of the methodological approach is given next for the multi-center models M3.

We retrieved inpatient hospitalization data on patients less than 18 years from January 2000 to December 2017 from 48 hospitals in the Cerner Health Facts Database. The data collected includes key information on each inpatient encounter, patient demographics, diagnosis, and medications. The 48 hospitals were selected based on a priori inclusion criteria including hospitals where the total number of inpatient encounters were greater than 2000 visits. These criteria ensure that we excluded potential noise data and that there is enough data to estimate model performance by hospital. We included multiple index visits and readmissions for individual patients and each visit that was itself a readmission was treated as an index visit for estimating subsequent readmission [22, 23].

We included variables previously identified to be predictors of readmission such as length of stay, admission type (elective, emergency, urgent, or trauma), diagnoses, and number of ED visits, hospitalizations, and previous 30-day readmissions within the prior 6 months of the visit [21, 35–37]. Demographics and proxies for socioeconomic status [38] such as age, gender, race/ethnicity, insurance payer/type, were also included. We searched for novel predictors by exploring the maximum previous length of stay within the last 6 months; an indicator for whether the index visit is itself a readmission from a previous visit; all generic medications a patient received as well as the route of administration during the hospitalization that met an a priori inclusion criteria. The a priori inclusion criterion for including a generic medication or medication administration route is based on frequency of administration. Only medications and medication administration routes for which at least 4% of hospitalized patients received or qualified were included to guarantee that there will be no problem of parameter estimation during model building due to rarity of event, and that only the most common medications likely to provide clinically significant opportunities for interventions are considered. Rare event studies require exact statistical tests that is beyond the scope of this study [39].

The dataset was randomly split into a training and test set with 50% of observations in each. The randomization limited distributional differences between the training and test set, and guided against overfitting. We built a machine learning model using random forest algorithm that can be used to easily explore complex non-linear relationships between a set of predictor variables and an outcome variable of interest while providing a measure of variable importance [40]. Rather than search a random parameter space, we chose to purposefully explore selected tree depths at a fixed number of trees with entropy as the impurity function [41]. We explored random forest modes with tree depths of 2 to 16 on a logarithm scale and fixed the number of trees at 256 based on previous studies [42]. We used 3-fold cross-validated AUC on the training data to determine the final/selected tree depth of the random forest model. We kept other parameters of the model (developed using Spark Machine Learning Library version 2.3.0) at their default value. In addition to the random forest model, we built an MLP model [43] by exploring 3-, 4-, 5- and 6-layer architectures where each hidden layer has half the neurons of the previous layer in a fully connected network. We used the sigmoid function for intermediate nodes and the softmax function on the output layer [44]. We used 3-fold cross validation for selection of the final MLP architecture/model. All data preprocessing and model development computation were carried out using the PySpark [28] (including Spark SQL and the Spark Machine Learning library [18, 45]) on the HealtheDataLab platform.

## Results

We successfully validated the HealtheDataLab platform by replicating the published study on readmission with similar results in performance and inference. Model M0 was shown to have an AUC of 0.79 by Ehwerhemuepha et al. [21] Models M1 and M2 were obtained using the best performing random forest model (obtained through cross validation) consisting of 256 trees and maximum tree depth of 16 on the HealtheIntent Data in the HealtheDataLab platform of adopting institution. The inclusion of patients with chemotherapy, increase in sample sizes, inclusion of medication classes, and use of the random forest architecture (as described in the Methods section) resulted in an AUC of 0.8226 for Model M1—an increase of 0.0326 in AUC over M0. Model M2 (which included both planned and unplanned readmissions in the training and test data) resulted in an AUC of 0.8756—an increase of 0.0530 in AUC over M1. The clinical significance of the improvement in AUC of model M2 over M1 is unknown since the data used to train model M2 included planned readmissions.

### Model M3 – the multi-center model for pediatric readmissions

There were 1.4 million pediatric encounters during the 18-year period considered in the multi-center database. Each hospital contributed different number of years of data with a mean of 13 years across all hospitals. Training and test set were divided in the same manner as the data used for models M1 and M2.

In the training data, there were 88,737 readmissions within the study period across all 48 hospitals in the training dataset with a readmission rate of 12.6%. There were 50.7% males, 44.4% females, and 4.9% patients with unknown sex. Patient coding for race/ethnicity included White (48.5%), African American/Black (22.6%), Hispanic (3.0%), Asian (1.5%), Native American (1.0%), and other/mixed/unknown races (23.4%). The mean (standard deviation) for patient age is 5.9 (6.0) years. There were 34.7% of patients with index visit length of stay of 4 days or more, 23.6% with a previous visit within the last 6 months, and 14.4% with maximum length of stay of 4 days or more from the previous visits. The percentage of patients who had a previous emergency department visit within the last 6 months of the index visit was 23.1%. We included an indicator for whether the index visit is itself a readmission from a previous visit, and there were 13.6% of such visits. Additionally, we included data on previous readmissions with 9.3% having had a readmission prior to, and not counting, the index visit. We considered diagnosis groupings as shown in Table 1, as well as generic medications and medication administration routes as shown in Table 2.

**Table 1:** Summary Statistics – Demographics, SES Proxies, Resource Utilizations, and Diagnosis

Variables	Level	Not Readmitted	Readmitted
		n (%) or mean (sd)	n (%) or mean (sd)
Gender	Male	311,260 (50.60)	45,338 (51.09)
	Female	273,009 (44.38)	39,194 (44.17)
	Unknown	30,861 (5.02)	4205 (4.74)
Race/ethnicity	White	304,559 (49.51)	36,807 (41.48)
	Asian	9205 (1.50)	1163 (1.31)
	Black/African American	144,376 (23.47)	14,833 (16.72)
	Native American	6537 (1.06)	688 (0.78)
	Hispanic	19,201 (3.12)	2120 (2.39)
	Others/Unknown	131,252 (21.34)	33,126 (37.33)
Age	–	5.73 (6.00)	6.90 (5.95)
Admission type	Emergency	276,802 (45.00)	27,986 (31.54)
	Elective	97,886 (15.91)	18,569 (20.93)
	Urgent	120,468 (19.58)	15,880 (17.90)
	Trauma	1521 (0.25)	51 (0.06)
	Others	118,453 (19.26)	26,251 (29.58)
Admission source	Referral	247,863 (40.29)	36,602 (41.25)
	Emergency/ER	105,515 (17.15)	9585 (10.80)
	Transfer	134,410 (21.85)	10,618 (11.97)
	Others	127,342 (20.70)	31,932 (35.98)
Length of stay	< 2 days	154,703 (25.15)	12,198 (13.75)
	< 4 days	268,635 (43.67)	24,353 (27.44)
	< 7 days	101,594 (16.52)	16,549 (18.65)
	7 days or more	90,198 (14.66)	35,637 (40.16)
Previous visits	0	506,358 (82.32)	31,389 (35.37)
	1	72,659 (11.81)	15,502 (17.47)
	2	18,949 (3.08)	8895 (10.02)
	3 or more	17,164 (2.79)	32,951 (37.13)
Previous maximum length of stay	< 2 days	524,932 (85.34)	36,463 (41.09)
	< 4 days	34,051 (5.54)	7349 (8.28)
	< 7 days	21,348 (3.47)	9670 (10.9)
	7 or more	34,799 (5.66)	35,255 (39.73)
Previous ED	0	478,460 (77.78)	62,745 (70.71)
	1	88,412 (14.37)	13,094 (14.76)
	2	27,999 (4.55)	5347 (6.03)
	3 or more	20,259 (3.29)	7551 (8.51)
Index visit is a readmission	Yes	52,355 (8.51)	43,633 (49.17)
	No	562,775 (91.49)	45,104 (50.83)
Readmission history	0	587,646 (95.53)	50,463 (56.87)
	1	14,808 (2.41)	8518 (9.60)
	2	5127 (0.83)	5456 (6.15)
	3 or more	7549 (1.23)	24,300 (27.38)
Diagnosis - ICD 10			
Bacterial infections	–	0.05 (0.27)	0.07 (0.39)
Blood and blood organs	–	0.11 (0.50)	0.25 (0.77)
Central Nervous System	–	0.20 (0.76)	0.25 (0.86)
Cerebrovascular blood vessels	–	0.05 (0.47)	0.06 (0.52)
Conditions from perinatal period	–	0.44 (1.98)	0.22 (1.52)
Congenital and chromosomal	–	0.31 (1.28)	0.36 (1.55)
Digestive	–	0.33 (1.23)	0.43 (1.40)
Ear, mastoid process	–	0.05 (0.35)	0.03 (0.29)
Endocrine	–	0.08 (0.56)	0.08 (0.58)
External causes of morbidity	–	0.10 (0.52)	0.07 (0.39)
Eye and adnexa	–	0.08 (0.80)	0.08 (0.77)
Genitourinary	–	0.15 (0.68)	0.15 (0.64)
Hypersensitivity	–	0.02 (0.19)	0.04 (0.28)
Immune mechanisms	–	0.01 (0.13)	0.02 (0.22)
Injury and poison	–	0.61 (8.44)	0.39 (5.45)
Ischemic heart disease	–	0.00 (0.04)	0.00 (0.04)
Malnutrition	–	0.01 (0.13)	0.02 (0.16)
Mental, behavioral, neurodevelopmental	–	0.26 (1.49)	0.22 (1.08)
Metabolic and other endocrine process	–	0.18 (0.67)	0.24 (0.92)
Musculoskeletal and connective tissues	–	0.12 (0.62)	0.12 (0.61)
Neoplasms	–	0.05 (0.32)	0.34 (0.87)
Other heart diseases	–	0.04 (0.38)	0.06 (0.47)
Overweight and hyperalimentation	–	0.01 (0.12)	0.01 (0.12)
Pregnancy, childbirth, puerperium	–	0.08 (1.02)	0.04 (0.74)
Pulmonary heart disease	–	0.03 (0.38)	0.04 (0.49)
Respiratory	–	0.48 (1.14)	0.35 (1.05)
Rheumatic fever	–	1.67 (0.02)	1.47 (0.01)
Rheumatic heart disease	–	0.00 (0.08)	0.00 (0.10)
Skin, subcutaneous tissues	–	0.14 (0.66)	0.11 (0.58)
Symptoms, signs, and abnormal lab findings	–	0.82 (1.71)	0.91 (1.94)
Viral infections	–	0.16 (0.79)	0.16 (0.80)
Health Hazards due to family/personal history	–	0.24 (0.79)	0.34 (0.98)
Health Hazards - others	–	0.26 (0.75)	0.21 (0.57)

**Table 2:** Summary Statistics – Medications

Variables	Not Readmitted	Readmitted
	mean (sd)	mean (sd)
Medication Administration Route/Type Count		
Inhalation	0.37 (0.99)	0.30 (0.91)
Injectable	2.14 (4.08)	2.61 (4.91)
Intramuscular	0.15 (0.44)	0.09 (0.41)
Intravenous	1.55 (3.18)	2.16 (4.31)
Ophthalmic	0.13 (0.44)	0.08 (0.41)
Oral	2.01 (3.27)	2.75 (4.42)
Rectal	0.14 (0.44)	0.11 (0.42)
Topical	0.31 (0.73)	0.38 (0.89)
Generic Medications		
Acetaminophen	0.43 (0.71)	0.39 (0.71)
Acetaminophen hydrocodone	0.05 (0.25)	0.04 (0.21)
Albuterol	0.18 (0.54)	0.13 (0.43)
Cefazoline	0.10 (0.38)	0.08 (0.33)
Ceftriaxone	0.07 (0.31)	0.07 (0.29)
Dexamethasone	0.08 (0.34)	0.11 (0.43)
Diphenhydramine	0.11 (0.39)	0.22 (0.53)
Docusate	0.04 (0.23)	0.06 (0.26)
Epinephrine	0.05 (0.25)	0.07 (0.30)
Erythromycin ophthalmic	0.07 (0.25)	0.02 (0.12)
Fentanyl	0.16 (0.45)	0.14 (0.45)
Glycopyrrolate	0.05 (0.24)	0.05 (0.24)
Heparin	0.17 (0.54)	0.33 (0.74)
Hepatitis B vaccine	0.06 (0.24)	0.01 (0.12)
Ibuprofen	0.14 (0.39)	0.09 (0.32)
Ketorolac	0.07 (0.29)	0.05 (0.24)
Lidocaine	0.08 (0.31)	0.07 (0.30)
Lidocaine topical	0.11 (0.33)	0.14 (0.36)
Lorazepam	0.07 (0.31)	0.14 (0.42)
LVP solution	0.48 (1.01)	0.72 (1.40)
LVP solution with potassium	0.20 (0.45)	0.21 (0.48)
Midazolam	0.12 (0.40)	0.12 (0.40)
Morphine	0.20 (0.56)	0.19 (0.58)
Ondansetron	0.20 (0.48)	0.29 (0.59)
Oxycodone	0.05 (0.24)	0.06 (0.28)
Phytonadione	0.08 (0.27)	0.03 (0.18)
Polyethylene glycol 3350	0.05 (0.23)	0.10 (0.30)
Potassium chloride	0.07 (0.32)	0.12 (0.43)
Propofol	0.10 (0.35)	0.10 (0.36)
Ranitidine	0.07 (0.30)	0.11 (0.37)
Rocuronium	0.05 (0.23)	0.04 (0.22)
Sodium chloride	0.19 (0.54)	0.25 (0.65)

**Fig. 2:**
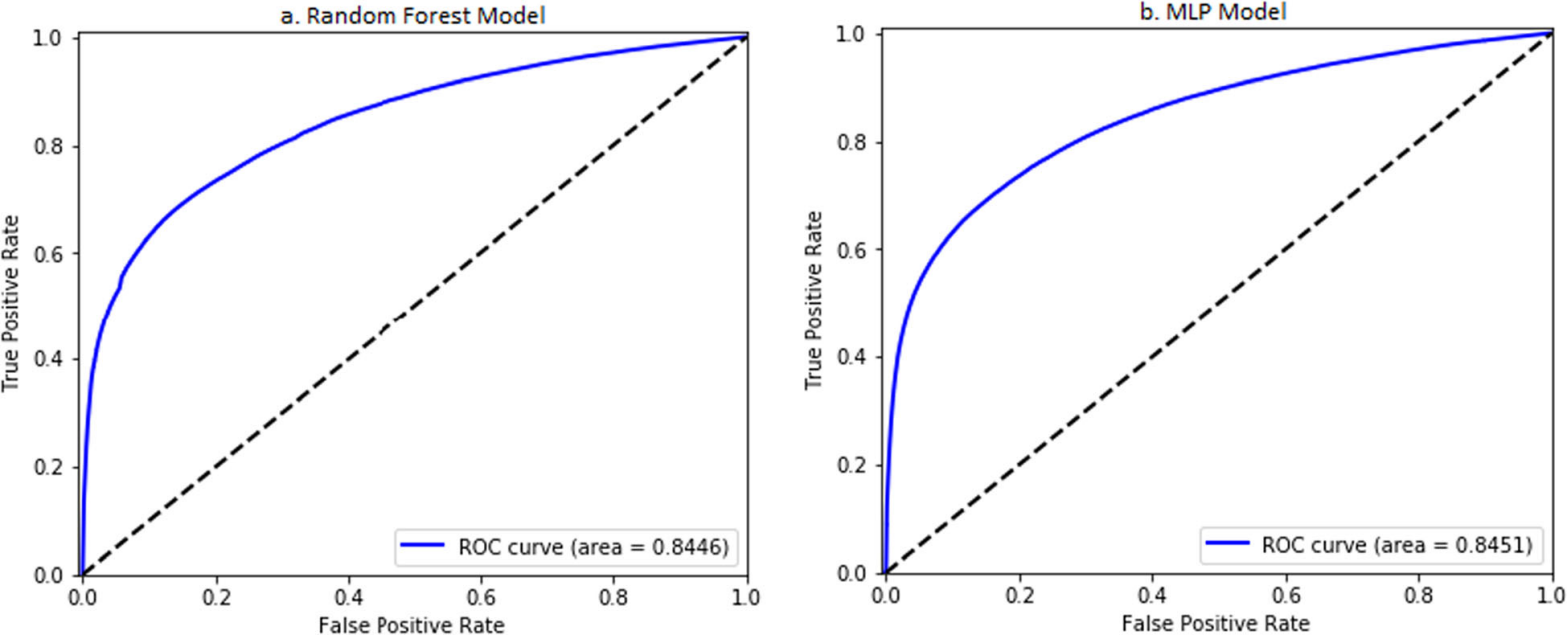
Area under the receiver operator characteristics of **a** the random forest and **b** the MLP models

**Fig. 3:**
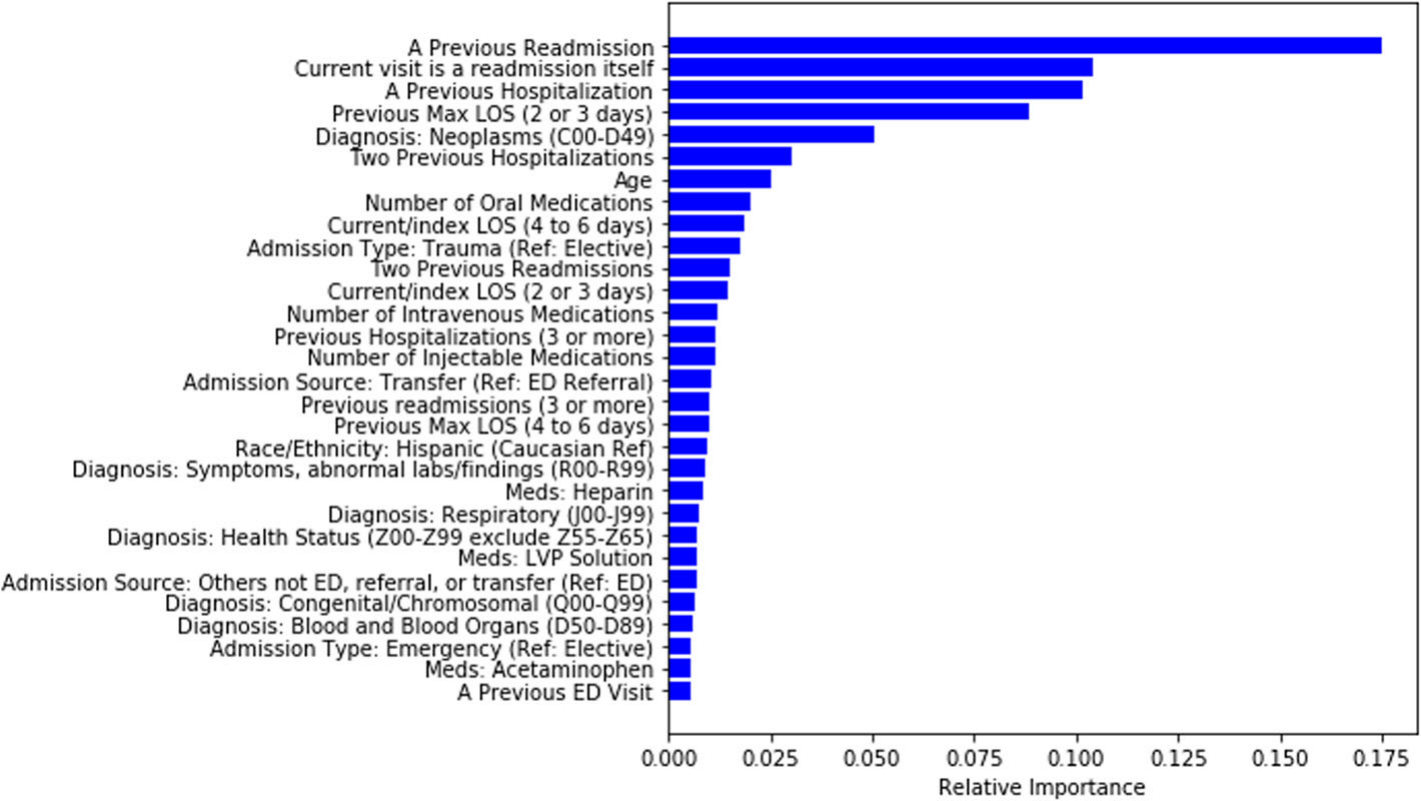
Top 30 important variables by the random forest model

**Fig. 4:**
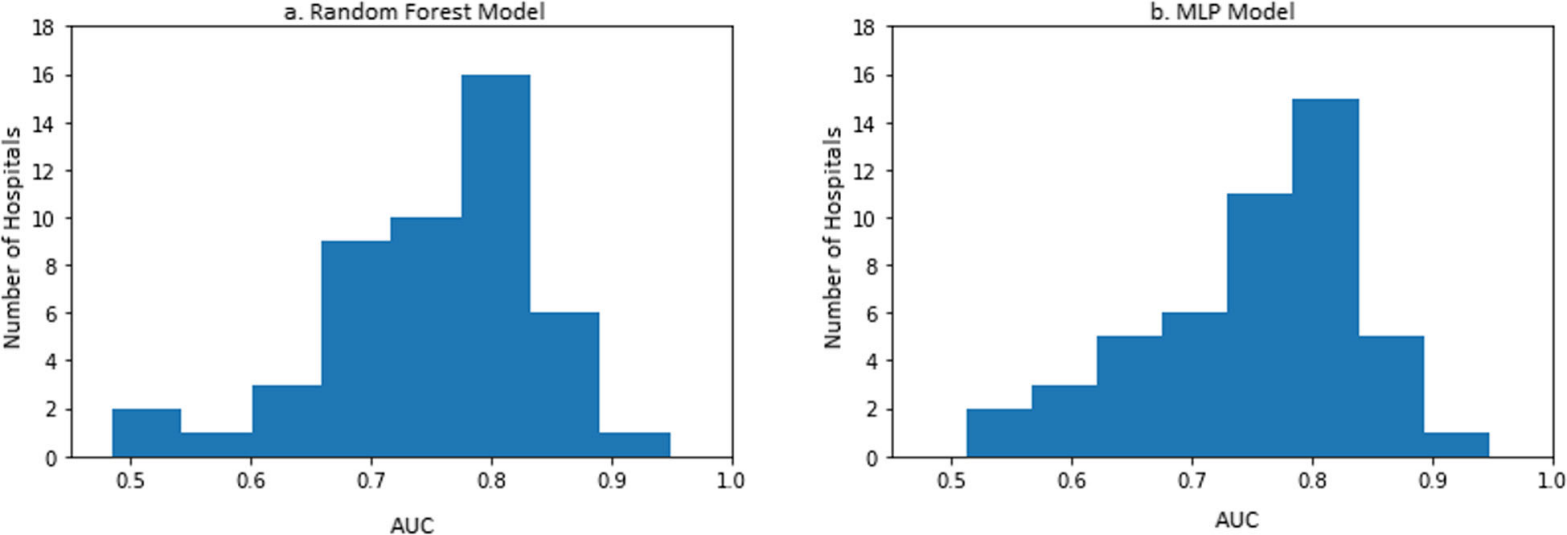
Performance distribution of model across all 48 hospitals

**Fig. 5:**
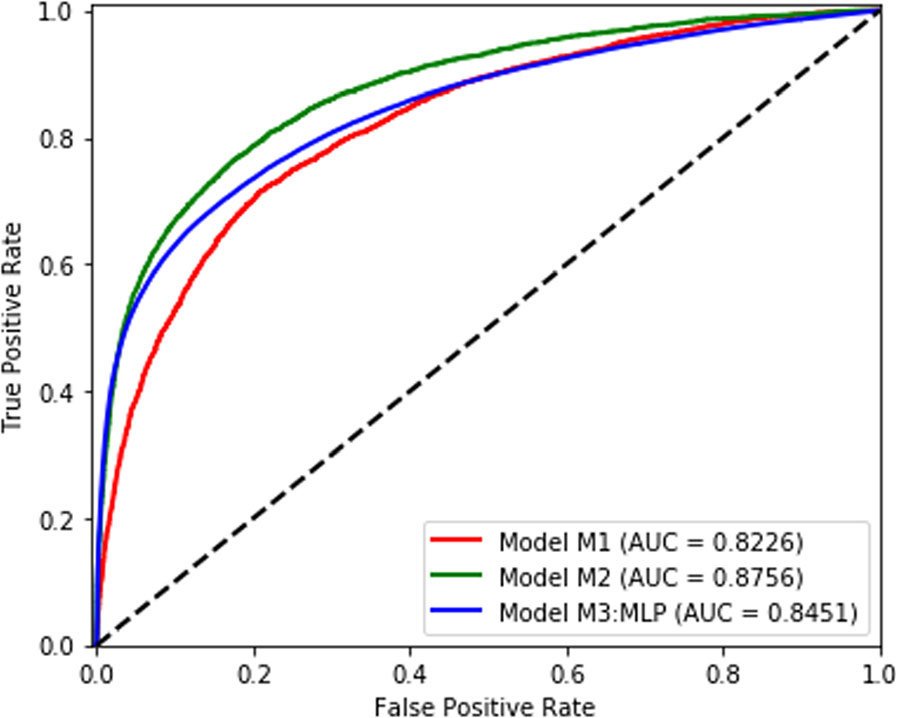
Comparison of random forest models of a single center unplanned readmission model (Model M1), a single center planned and unplanned readmission model (Model M2), and a multi-center MLP model of readmission (Model M3:MLP)

The resulting random forest model (M3:RF) consisted of 256 trees with a tree depth of 16. There was a linear increase in the training data cross-validated AUC as the depth of tree increased exponentially. The AUC of the model on the test set is 0.8446 (0.8444, 0.8447) across all 48 hospitals (Fig. 2a). The top 30 most important variables are shown in Fig. 3. The most important variables include previous readmissions, index visits that are readmissions from a prior visit, previous hospitalizations, and the length of stay of visits prior to the index visit. The performance of model M3:RF across each hospital was variable with majority of hospitals having AUCs greater than 0.75 as shown in Fig. 4a. The best performing MLP model (M3:MLP) consists of 4 layers and resulted in an AUC of 0.8451 (0.8449, 0.8453) across all 48 hospitals (Fig. 2b). The distribution of AUCs across the hospitals (Fig. 4b) was identical to M3:RF but with less spread in this occasion (that is, conditional on the hyperparameter search space we explored in this study). In Fig. 5, we presented a comparison of the random forest model performance for models M1, M2, and M3.

#### Comparison with models from previous studies

We conducted head-to-head model performance comparisons with the LACE readmissions model. The LACE readmissions model is a model that uses 4 variables (Length of stay, acuity of admission, comorbidity of the patient, and emergency department use) as predictors of readmissions. The AUC of the corresponding LACE model equivalent of M1 is 0.6952 (0.6851, 0.7054); the AUC of its equivalent of M2 is 0.6786 (0.6705, 0.6867); and the AUC of its equivalent of M3 is 0.7014 (0.6994, 0.7033). In other words, all models developed in this study have higher model performance than the standard LACE readmission model. In addition to these comparisons to the LACE readmission model, we searched for model performance of similar models using a systematic review of over 30 studies and 26 unique models [24] and an additional review of 60 studies [46] with 73 unique models. This indicate that the AUCs for general medicine (all cause) prediction models ranged from a minimum of 0.56 to a study with a maximum of 0.83 in models for pediatrics or comparable in population to the pediatric models herein. These values for the model performance indicate that the models presented in this study (M2 and M3) have significant improvements over existing pediatric models.

## Discussion

We provided an architectural layout of the design of Cerner’s HealtheDataLab platform, which is an advanced analytics platform for parallel distributed data science using cloud computing. This study can help other healthcare organizations replicate the platform using any cloud provider. However, highly specialized skills different from those of a data scientist and statistician are required to appropriately provision and manage such tools – in fact, this is a domain for data engineers. The main contribution of HealtheDataLab is the utilization of high-performance cloud computing resources for seamless integration with and update from the EMR. Therefore, structured EMR data is readily accessible in a high-performance cloud platform wherein open source tools such as Apache Spark can be used for analyses of any size or complexity. The tool is managed by data engineering teams at Cerner and made for teams with people who are highly skilled in data science as well as in big data analysis. As a result, it does not lend itself to use by administrators or teams without the technical skillsets of a data scientist. On the one hand, the contribution of HealtheDataLab implies that advanced parallel distributed analytics can be run on EMR data in search for associations that may be useful for advancement of care. On the other hand, highly skilled data scientists or statisticians are required to use this tool which is inaccessible to administrators or providers without in-depth big data and data science skills. In addition to EMR data, other data sources (such as the Health Facts Database) can be added directly to an Amazon S3 bucket which is accessible by the platform. We validated the platform by replicating a previous study on a novel model for unplanned readmission with a reported AUC of 0.79 using the EMR of the corresponding institution of study.

We provided an application of the tool by developing a multi-center random forest and multi-layer perceptron (neural network) models for predicting all cause planned and unplanned readmission with an AUC of approximately 0.8446 and 0.8451, respectively. The ranking of predictor importance by the random forest model indicates that the numbers of certain medications administered and the routes of administration (during hospitalization) are associated with the risk of readmission. Novel variables, such as the maximum length of stay within the last 6 months (excluding index visit) and an indicator representing whether the index visit is a readmission itself, were found to be important predictors. We showed that in all cases, the models developed here were more powerful than the LACE readmission model, competitive with all other models developed in literature, and had the highest AUC in pediatric readmission studies.

This multi-center application is limited by use of deidentified data for which unplanned visits cannot be separated from planned ones. On the one hand, if there is no significant difference between risk factors for planned and unplanned readmissions, a model considering all readmissions would achieve statistical boost over unplanned readmissions due to increase in the readmission rate/number of cases in the training set. On the other hand, if there are significant differences between both groups, the accuracy of predicting unplanned readmission using models on all readmission may result in decreased performance of the model. The strength from large sample sizes and use of multi-center EMR data helps to reduce concerns about differences between planned and unplanned readmissions. Further studies using logistic regression with random intercepts (to account for baseline differences between hospitals) are required to determine effect sizes and their directions, and statistical or clinical significance. The list of the top 30 predictors identified in this work provides organizations additional variables to consider during model development. This may result in reducing false positive predictions that waste clinical intervention efforts. Improvements in such models put limited resources to better use for providing in-hospital interventions [21] as well as post-discharge follow-up. The machine learning methodology used in this study may also be used by other organizations although our study indicates that performance of predictive models of readmission may vary across pediatric institutions—this merit further study.

## Conclusion

We have demonstrated the utility and strength of high-performance cloud computing resources, directly integrated to the EMR, for advanced predictive analytics in healthcare. In addition to the analysis of our institution’s readmission data, we were able to apply machine learning algorithms on data from multiple institutions originating outside of our EMR. Therefore, this study provides information required by organizations considering high-performance cloud computing resources, as well as information by which they can improve predictive models for readmission. Most notably, we have demonstrated that cloud computing solutions that integrate directly with the electronic medical records can be developed for application of data science algorithms in building and deploying predictive models back to the EMR. More specifically, we addressed the 3 goals of this study as follows: First, researchers in healthcare have applied cloud computing for individual projects by individual teams to a select problem and dataset within hospitals. HealtheDataLab provides an enterprise-wide approach for storage of all EMR data and the tools/algorithms required to analyze the data for research and for business intelligence applications. Second, HealtheDataLab can be seamlessly and directly integrated with the EMR and receive automated updates from it. Lastly, machine and artificial learning models can be developed and used for discovery of novel risk factors as well as improvement of existing models. In this study, we did not show how models can be automatically deployed from HealtheDataLab to the EMR for use as a decision support tool as the functionality was still nascent at the time of writing. Although we used HealtheDataLab in this study, the goal is to demonstrate that tools like it (regardless of EMR and cloud computing vendors) can be developed and used to further the use of big data analytics and data science in healthcare. Managed services and tools such as HealtheDataLab would ensure that cloud computing in medicine is accessible to a wider number of teams who, otherwise, may not be able to navigate and manage the highly technical ecosystem of cloud computing tools.

## Supplementary information

**Additional file 1.** Amazon Web Services Cloud Resources Used.

## Data Availability

The Cerner Health Facts Database (now referred to as the Cerner Real World Data) is available to researchers at contributing hospitals (and their research affiliates) upon request made directly to Cerner Corporation. Data on patients from the corresponding pediatric hospital is available only upon request and approval of the Institutional Review Board of the hospital.

## Abbreviations

AUC: Area under the receiver operator characteristic curve
AWS: Amazon web services
EC2: Amazon elastic compute cloud
EHR: Electronic health records
EMR: Electronic medical records
HIPAA: Health insurance portability and accountability act of 1996
IRB: Institutional review board
MLP: Multilayer perceptron neural network algorithm
RDS: Amazon relational database service
RF: Random forest algorithm
S3: Amazon simple storage solution
VPC: Amazon virtual private cloud
