# Supplementary material for: HealtheDataLab – a cloud computing solution for data science and advanced analytics in healthcare with application to predicting multi-center pediatric readmissions
Source: BMC Med Inform Decis Mak. 2020 Jun 19;20:115. doi: 10.1186/s12911-020-01153-7 (PMC7304122; doi:10.1186/s12911-020-01153-7)
Supplement: Supplementary file 1 — Additional file 1. Amazon Web Services Cloud Resources Used. [file 12911_2020_1153_MOESM1_ESM.docx]

**Appendix 1. AWS Services Used**

|  |  |
| --- | --- |
| Service Name | Description |
| CloudFormation | Provides a common language for you to describe and provision all the infrastructure resources in your cloud environment. HealtheDataLab utilizes it to provide a template to spin up the user’s environment. |
| Identity and Access Management (IAM) | Enables users to manage access to AWS services and resources securely. HealtheDataLab utilizes IAM to create users and groups with permissions to allow or deny access to resources within the environment. |
| DataPipeline | A web service that helps users reliably process and move data between different AWS compute and storage services at specified intervals. HealtheDataLab leverages Data Pipeline to detect the arrival of new data from HealtheIntent. |
| Simple Notification Service (SNS) | A messaging service that enables users to send and receive notifications regarding other microservices within their AWS environment. HealtheDataLab leverages SNS to send notifications in case jobs launched by the Data Pipeline fail. |
| Virtual Private Cloud (VPC) | Allows users to provision a logically isolated section of the AWS cloud where they can launch AWS resources in a virtual network they define. HealtheDataLab leverages this to restrict access to only those users with permission to access the environment. |
| Elastic Compute Cloud (EC2) | A web service that provides secure, resizable compute capacity in the cloud. HealtheDataLab leverages EC2 to run JupyterHub, which drives the user experience within HealtheDataLab. |
| Elastic Map Reduce (EMR) | A managed Hadoop framework that makes it easy, fast, and cost-effective to process vast amounts of data across dynamically scalable Amazon EC2 instances. HealtheDataLab leverages EMR to scale the EC2 instance compute capacity to improve performance in the testing and training of machine learning algorithms. |
| Relational Database Service (RDS) | Makes it easy to set up, operate, and scale a relational database in the cloud. HealtheDataLab leverages RDS to back a Hive Metastore which maintains metadata for virtual databases and tabular representations of data available in S3. |
| Simple Storage Service (S3) | Object storage built to store and retrieve any amount of data from anywhere. HealtheDataLab leverages S3 to storage system configuration files, host data and Jupyter Notebooks, and hold a history of system logs. |
| CloudWatch Logs | Used to monitor, store, and access log files from AWS services such as EC2, CloudTrail, or Route 53. HealtheDataLab uses CloudWatch to aggregate and store logs streamed from the EC2 instances within the environment. |
| Key Management Service (KMS) | A managed service that makes it easy for you to create and control the encryption keys used to encrypt your data. HealtheDataLab leverages this service to maintain AES-256 encryption on all data both at-rest and in-transit. |
| Lambda | Allows users to run code without provision or managing services. HealtheDataLab leverages Lambda in the automatic tear-down of the environment. |
